# Supplementary material for: End Sequence Analysis Toolkit (ESAT) expands the extractable information from single-cell RNA-seq data
Source: Genome Res. 2016 Oct;26(10):1397–410. doi: 10.1101/gr.207902.116 (PMC5052061; doi:10.1101/gr.207902.116)
Supplement: Supplemental Material [file supp_gr.207902.116_Supp_Materials.tar.gz › ESAT-esat_v0.1/doc/html/classjaligner_1_1matrix_1_1_matrix.html]

ESAT: jaligner.matrix.Matrix Class Reference


ESAT


- Main Page
- Related Pages
- Packages
- Classes
- Files

- Class List
- Class Index
- Class Hierarchy
- Class Members

All Classes Namespaces Files Functions Variables Enumerator Pages

- jaligner
- matrix
- Matrix

Public Member Functions |
Static Public Attributes |
List of all members

jaligner.matrix.Matrix Class Reference

Inheritance diagram for jaligner.matrix.Matrix:

[legend]

Collaboration diagram for jaligner.matrix.Matrix:

[legend]

|  |  |
| --- | --- |
| Public Member Functions | |
|  | Matrix (String id, float[][] scores) |
|  | |
| String | getId () |
|  | |
| float[][] | getScores () |
|  | |
| float | getScore (char a, char b) |
|  | |

|  |  |
| --- | --- |
| Static Public Attributes | |
| static final int | SIZE = 127 |
|  | |

## Detailed Description

Scoring matrix.

Author
:   Ahmed Moustafa (ahmed.nosp@m.@use.nosp@m.rs.sf.nosp@m..net)

## Constructor & Destructor Documentation

|  |  |  |  |
| --- | --- | --- | --- |
| jaligner.matrix.Matrix.Matrix | ( | String | *id*, |
|  |  | float | *scores*[][] |
|  | ) |  |  |

## Member Function Documentation

|  |  |  |  |  |
| --- | --- | --- | --- | --- |
| String jaligner.matrix.Matrix.getId | ( |  | ) |  |

Returns
:   Returns the id.

Here is the caller graph for this function:

|  |  |  |  |
| --- | --- | --- | --- |
| float jaligner.matrix.Matrix.getScore | ( | char | *a*, |
|  |  | char | *b* |
|  | ) |  |  |

Parameters
:   |  |  |
    | --- | --- |
    | a |  |
    | b |  |

Returns
:   score

|  |  |  |  |  |
| --- | --- | --- | --- | --- |
| float [][] jaligner.matrix.Matrix.getScores | ( |  | ) |  |

Returns
:   Returns the scores.

## Member Data Documentation

final int jaligner.matrix.Matrix.SIZE = 127

static

The size of the scoring matrix. It is the number of the characters in the ASCII table. It is more than the 20 amino acids just to save the processing time of the mapping.

---

The documentation for this class was generated from the following file:

- java/jaligner/matrix/Matrix.java


---

Generated on Fri Aug 15 2014 15:11:06 for ESAT by  

 1.8.7
